# Delayed breast cancer relapse with pleural metastasis and malignant pleural effusion after long periods of disease‐free survival

**DOI:** 10.1002/rcr2.375

**Published:** 2018-10-26

**Authors:** Antony Divananth Rawindraraj, Christine Yang Zhou, Vikas Pathak

**Affiliations:** ^1^ Department of Internal Medicine Campbell University School of Osteopathic Medicine Lillington NC USA; ^2^ Department of Pulmonary and Critical Care Medicine WakeMed Health and Hospitals Raleigh NC USA

**Keywords:** Breast cancer relapse, breast cancer metastasis, malignant pleural effusion, pleural effusion

## Abstract

Breast cancer relapse remains a common cause of morbidity and mortality in patients who undergo initial treatment with surgery and with or without concurrent chemotherapy or radiation. Relapse rates remain high within the first decade after initial treatment, after which the risk of relapse decreases. While common within the first year of breast cancer diagnosis, pleural metastasis with malignant pleural effusion (MPE) after 10–12 years of a disease‐free period is rare. Here, we present two uncommon cases of delayed breast cancer relapses with pleural metastasis, which caused MPEs.

## Introduction

Breast cancer is the most common leading cause of cancer‐related death among women. While it carries a high risk of local and distant relapse or metastasis during the first decade after initial treatment, delayed relapse of breast cancer after 10 years of disease‐free survival (DFS) is rare 1. The most common sites of breast cancer relapse are bone (70.6%) followed by liver (54.5%) and lung (31.4%) 2. In addition, pleural metastasis of breast cancer commonly occurs during the initial few years following breast cancer diagnosis, but it is uncommon after a prolonged disease‐free period 3. Pleural metastasis of breast cancer often presents with malignant pleural effusion (MPE), which leads to recurrent attacks of dyspnoea and frequent hospital visits for surgical drainage 4.

Here, we present two cases of breast cancer relapses after 21 and 14 years of DFS and MPE secondary to pleural metastasis.

## Case Report

### Case 1

**Figure 1:**
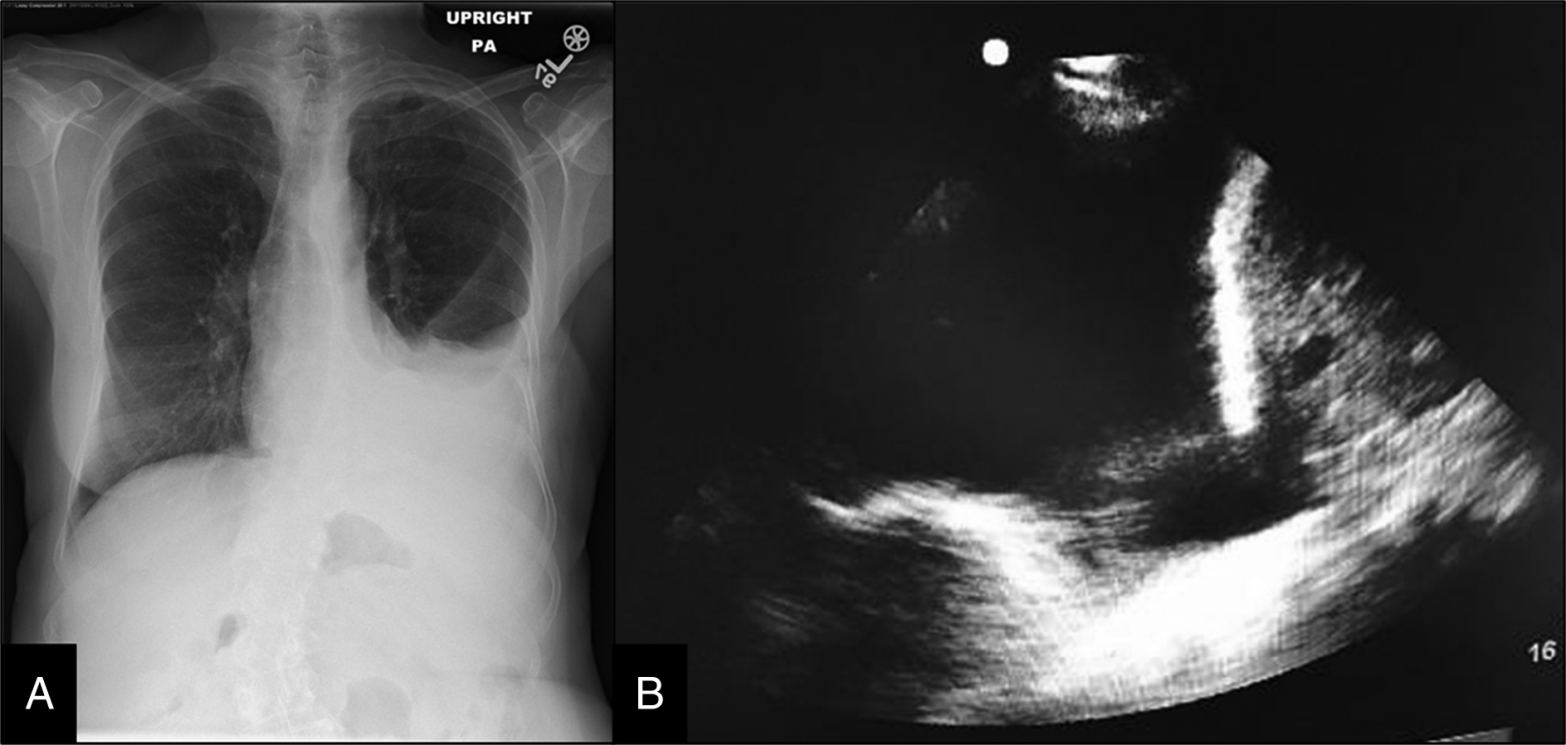
(a) Chest X‐ray demonstrating left‐sided pleural effusion. (b) Ultrasound of the left thorax showing pleural effusion.

A 74‐year‐old female presented to her cardiologist with a 6‐week history of worsening dyspnoea on exertion. She had a past medical history of stage IIIa invasive lobular carcinoma of the left breast, positive for both oestrogen and progesterone receptors (oestrogen receptor (ER)/progesterone receptor (PR)), which was diagnosed 21 years back. At that time, she had undergone modified radical left mastectomy at age 53, followed by treatment with adjuvant chemotherapy and radiation. Due to her current symptoms of dyspnoea, a chest X‐ray was taken, which showed moderate‐sized left pleural effusion compared to a chest X‐ray from 2 years ago (Fig. 1a). She was therefore referred to Interventional Pulmonology for further workup of pleural effusion. An ultrasound‐guided left‐sided thoracentesis demonstrated lymphocyte‐predominant exudative pleural effusion (Fig. 1b). Cytology of the pleural fluid showed malignant cells with immunohistochemistry positive for breast tumour markers. Analysis of the pleural fluid was weakly positive for ER and PR and negative for human epidermal growth factor 2 (HER2) receptors. A post‐thoracentesis chest computerized tomography scan with contrast showed a small residual left pleural effusion with a right pulmonary nodule. The patient was diagnosed with metastatic breast carcinoma and was referred to Oncology for further treatment.

### Case 2

A 76‐year‐old female presented to her cardiologist with a 3‐week history of progressive shortness of breath on exertion. Her cardiac workup was negative, including an ejection fraction of 60–65% on echocardiography. However, chest X‐ray showed right‐sided pleural effusion. She was therefore sent to Interventional Pulmonology for further workup. Further review of her past medical history indicated that, at age 62, she was diagnosed with invasive ductal carcinoma of the left breast as well as in‐situ ductal and lobular carcinoma of the right breast. At that time, she underwent bilateral simple mastectomies with adjuvant chemotherapy. A right‐sided thoracentesis was performed and demonstrated lymphocyte‐predominant exudative pleural effusion. Cytology of the pleural fluid was negative for malignancy.

**Figure 2:**
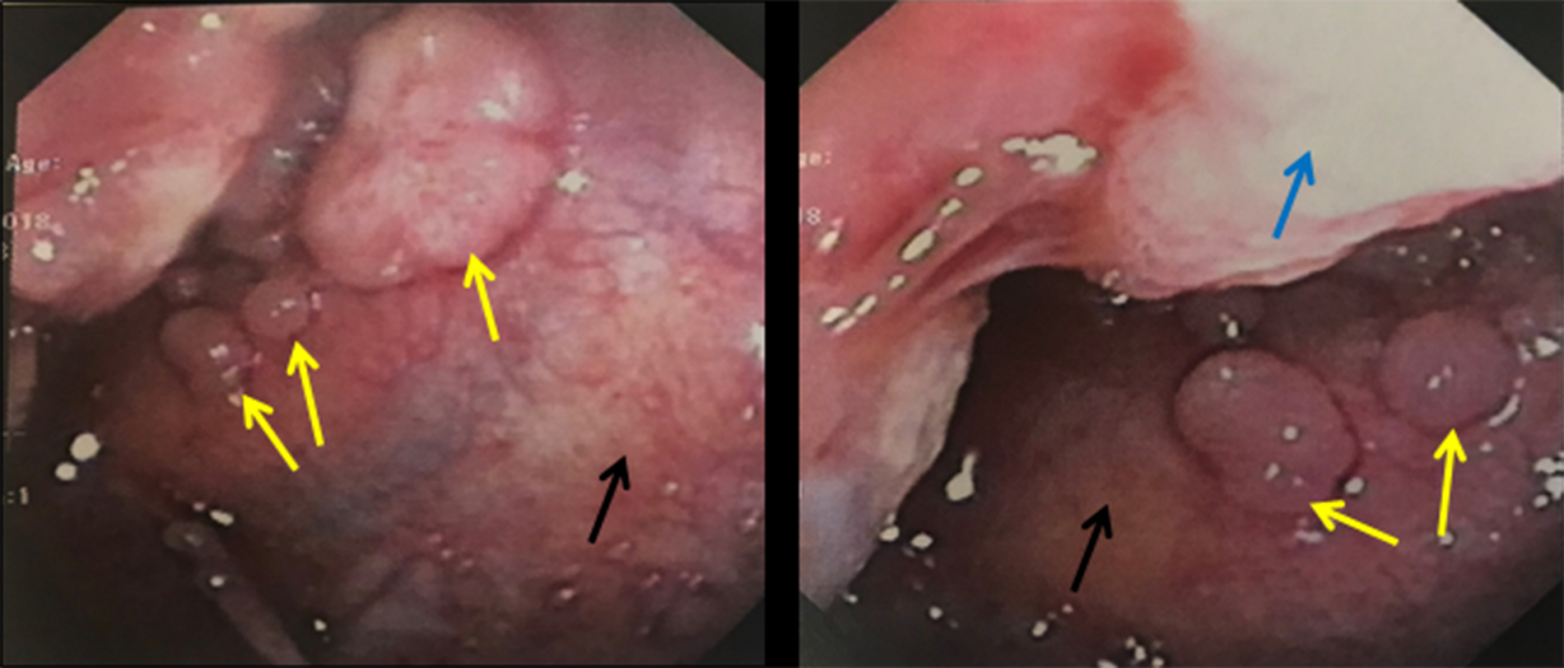
Pleuroscopy showing multiple masses (yellow arrows) of various sizes originating from the parietal pleura (black arrows). Blue arrow: Right lung.

Over the next few weeks, the patient developed progressively worsening dyspnoea. A repeat chest X‐ray 4 weeks following her initial thoracentesis showed recurrent right‐sided pleural effusion. Pleuroscopy with pleural biopsy was scheduled. Pleuroscopy showed multiple masses throughout the parietal pleura (Fig. 2), diaphragm, and on the right lung. Biopsy of the pleural masses showed metastatic adenocarcinoma positive for breast markers and negative for lung or gastrointestinal markers. She was ultimately diagnosed with MPE secondary to metastatic ER+/PR+/HER2—breast carcinoma. She was referred to Oncology for further treatment.

## Discussion

These case reports demonstrate two incidences of delayed breast cancer relapse that occurred 21 and 14 years after initial treatment. Typically, breast cancer has a dual peaked relapse pattern, with most relapses occurring during the second and sixth to ninth years after initial treatment 1. A case series by Omidvari et al. in 2013 demonstrated six cases of delayed breast cancer relapses with significantly increased years of DFS after initial treatment. At the time of breast cancer diagnosis, all six patients were treated with mastectomies with or without adjuvant chemotherapy or radiation therapy. One of the patients experienced breast cancer relapse and metastasis to the cervical lymph nodes after 32 years of DFS. This is by far the longest reported delay in breast cancer relapse to the best of our knowledge 1. Along with the cases presented by Omidvari et al., patients in our case reports showcase the continued risk of relapse far beyond the expected time period. These cases highlight the importance of a high degree of suspicion when approaching patients with a history of malignancy regardless of how many years they have been disease free. However, the difference between the case series between Omidvari et al. and our cases is the metastasis to pleural space.

In addition to delayed breast cancer relapse, these two patients also exhibit incidences of MPE secondary to pleural metastasis of breast cancer. Breast cancer relapse and metastasis most commonly occur in the bone, liver, or lungs, especially with the ER+/HER2 breast cancer subtype 2. Even though pleural metastasis is common in patients who were recently diagnosed with breast cancer 5, it is a much rarer phenomenon in patients with prolonged DFS. A case series conducted by Basavaraj et al. in 2015 reported that recurrent MPE was observed in nine patients with breast cancer. In every patient, MPE occurred within one year of their initial diagnosis of breast cancer 5. In 2013, Shinohara et al. presented a patient with breast cancer status post‐mastectomy and adjuvant hormonal therapy who was diagnosed with pleural metastasis of breast cancer and MPE after 12 years of DFS 3. Similar to Shinohara et al., both patients in our cases show the possibility of pleural metastasis of breast cancer relapse leading to MPE even after an extended period of DFS.

A diagnosis of MPE carries a poor prognosis with short expected time of survival and poor quality of life 6. The survival time after MPE diagnosis secondary to all known metastatic cancers ranges from one month to eight years 7. However, the median survival of MPE secondary to breast cancer metastasis is 15 months following pleural fluid accumulation 7. Due to the poor prognosis, more invasive treatments, such as pleurectomy, or treatments with major adverse side effects, like chemotherapy, are usually not considered. Thus, the most common management of MPE in patients with breast cancer relapse is repetitive thoracentesis. Other treatment options that may be considered for MPE are placement of an indwelling pleural catheter or talc pleurodiesis 6. These procedures usually provide symptomatic relief from dyspnoea through continued drainage of fluid, allowing for improved pulmonary function. However, they rarely decrease the morbidity and mortality associated with MPE caused by breast cancer metastasis 3.

Delayed presentation of MPE due to pleural metastasis is a rare occurrence in breast cancer relapses, especially after prolonged DFS. It is associated with limited treatment options and reduced survival time. Optimal patient care requires a high degree of suspicion for MPE regardless of how long the patient has been disease free.

### Disclosure Statement

Appropriate written informed consent was obtained for publication of this case report and accompanying images.
